# Co-creation of staff training to address health-related social needs in emergencies

**DOI:** 10.3389/fpubh.2025.1441368

**Published:** 2025-04-09

**Authors:** Linda Highfield, Gayla Ferguson

**Affiliations:** ^1^Department of Medicine, Baylor Scott and White Health System, Temple, TX, United States; ^2^Department of Management, Policy and Community Health Practice, UTHealth Science Center at Houston School of Public Health, Houston, TX, United States

**Keywords:** adaptive training, patient navigation, health related social needs, public health emergency, AHC model

## Abstract

**Background:**

Health-related social needs (HRSN), such as housing and transportation barriers, contribute to poor health outcomes and increased healthcare costs. Patient navigators help connect patients to community resources, but workforce training gaps are a challenge. The Strengthening Peer AHC Navigation (SPAN) study aimed to enhance navigation training during the COVID-19 Public Health Emergency.

**Methods:**

Using a stakeholder-driven peer planning approach, SPAN co-developed a quality improvement plan for patient navigation training. Training focused on housing and transportation navigation and included didactic sessions, hands-on case studies, and biweekly expert consultations. Self-efficacy surveys measured navigator confidence pre- and post-training, and changes in navigation case resolution rates were assessed.

**Results:**

Seventeen navigators participated in training. Self-efficacy scores improved, particularly in housing and transportation navigation. Resolved navigation cases increased by 29% (*p* = 0.001) over 6 months post-training. Participants reported increased confidence, knowledge, and empathy for patients with HRSN.

**Conclusion:**

The SPAN peer planning model successfully developed and implemented an adaptive navigation training program, improving navigator confidence and patient outcomes. Findings highlight the value of stakeholder-driven training and ongoing expert support in strengthening the social needs workforce. Further research should explore sustainable models for workforce development in healthcare settings.

## Introduction

Health-related social needs (HRSN) including food insecurity, housing instability, transportation, and difficulty paying bills are associated with a range of poor health outcomes, increased healthcare utilization and cost (1–6). HRSN represent a health equity issue with a disproportionate burden on under, and uninsured, minority patients (2). These patients often seek care in Emergency Departments (EDs) due to insufficient access to healthcare coupled with their HRSN and other risk factors (2). To address HRSN, and improve healthcare utilization and cost, patient navigators play a pivotal role often serving as link workers who provide patient education and facilitate a connection to community resources to address identified HRSN (1, 6). The largest test of HRSN programs with link workers in the US was the CMS Accountable Health Communities Model (AHC) which tested systematic HRSN screening, referral, patient navigation (Assistance Track) and community engagement (Alignment Track) for Medicare, Medicaid and dually covered beneficiaries (7). In Assistance, screening via the AHC screening tool, referral to community organizations and patient navigation was conducted using a randomized controlled trial design (RCT) (2, 8). Alignment added community advisory boards and continuous quality improvement, with the goal of aligning community resources to resolve HRSNs but was not an RCT. In both Tracks, Bridge Organizations (BO) served as the anchor or hub and led Model activities with clinical delivery sites (hospital emergency departments, labor and delivery departments and ambulatory clinics). BO were a range of organization types including health systems, public health, academic and non-profit organizations. 28 BOs supported 186 screeners and 159 navigators employed by the grant. “More than 1 million (1,114,099) unique beneficiaries were screened between May 2018 and January 2023. Of those, 18% (204,447) were eligible for navigation services (one or more core HRSNs and two or more self-reported ED visits in the 12 months prior to their screening)” (9). The AHC Model required implementation strategies be used by BOs and included a mixed methods evaluation by the external evaluator for the overall Model. BO staff training was a requirement for patient navigators; however, BOs were able to develop and implement their own training programs, leading to potential variability in content, modality, and overall quality of the workforce training provided (10, 11). The National Academies of Sciences, Engineering, and Medicine (NASEM) report on addressing HRSN in healthcare settings also indicated there is a current lack of data on the type of staff serving as link workers, and the workforce training that they may have received specific to assessing and addressing HRSN in the US (12). In short, to successfully connect patients to community resources, patient navigators require education and training that is often beyond the scope of existing community health worker (CHW) or patient navigator training programs in the United States.

The public health emergency (PHE) caused by the novel Coronavirus (COVID-19) represented a pivotal time for healthcare, HRSN, and patient navigators serving as the link between patients and community resources. While initially disrupting and reducing healthcare and ED utilization, as the pandemic progressed, EDs again became a focal point of patient contact with some studies noting rebounds in ED visits particularly for minority African American patients by April to May 2020 in safety net hospitals (13). Patient navigators working in healthcare settings, including EDs, were sometimes removed from working in person on patient units during the pandemic to reduce unnecessary contact and disease spread. In addition, HRSN increased substantially during the pandemic, especially food insecurity, housing instability and transportation needs (14–16). This enormous increase in need was coupled with strains on available community resources such as food pantries, housing assistance programs, and even public transit agencies who reduced or shifted their operations in response to the pandemic (17, 18). Many community agencies, and even local government funded programs dealt with challenges from reduced revenue resulting in difficulty maintaining operations at pre-pandemic levels (18). In short, the PHE created a challenging and dynamic situation where more patients needed support for HRSN but faced reduced access to both healthcare and community resources. This was coupled with reduced access to patient navigators who were often forced to work remotely and had to find innovative ways to continue to provide their education and facilitation services to patients. Little is currently known about patient navigator training programs for HRSN in the US, and even less is known about how programs were created or adapted for the PHE to respond to evolving patient and navigator needs and rapidly changing availability of community resources.

The Strengthening Peer AHC Navigation (SPAN) study, was conceptualized to structure technical assistance to AHC BO and CDS who had identified gaps in the implementation of the AHC Model, particularly within their provision of patient navigation services (19). Using an integrated framework blending the Consolidated Framework for Implementation Research, Intervention Mapping, and the Expert Recommendations for Implementing Change compilation (ERIC), a comprehensive technical assistance protocol was created to help Bridge Organizations improve delivery of the AHC Model (20–22). Using these three implementation science frameworks, SPAN applied four succinct steps, assessment, planning, implementation with technical assistance, and evaluation. A mixed methods assessment was conducted to understand baseline implementation processes, identified needs, and readiness for change and results have been previously reported (7, 19). Briefly, SPAN’s assessment found specific workforce training gaps for navigators in the AHC Model, including patient engagement, communication, knowledge, and boundary spanning skills needed to successfully connect patients to community resources, particularly for housing and transportation needs (7, 23). Our objective in this study is to describe the pilot test of the SPAN peer planning process and the development and implementation of a quality improvement plan focused on patient navigation training and workforce development for HRSN during the PHE at one AHC BO and CDS located in Houston, Texas.

### Setting

The BO and partnering clinical delivery sites in this study were in the AHC Model Assistance Track in Houston, Texas. They had previously co-created the implementation strategy for AHC (1). The SPAN stakeholder and peer planning method used in this study was to co-create a quality improvement (QI) plan during implementation of the AHC Model to enhance navigation service delivery. The QI plan included adaptive booster training for HRSN patient navigation for housing instability and transportation needs during the PHE which were identified as a key gap area in our largest partnering safety net hospital system’s existing emergency room patient navigation program. The patient navigation program in this study had been operating for more than 20 years using CHWs as patient navigators. Navigators and Managers were Texas certified CHWs and received regular continuing education as required to maintain Texas certification (24). CHW certification in Texas is a generalist model with a set of eight core competencies (24, 25). As part of our AHC Model implementation, AHC assigned navigators received Model specific training prior to implementation which included both didactic and hands-on training in HRSN, screening, referral and navigation. Navigation training included active listening, action planning and behavior change techniques including role modeling and vicarious reinforcement (1). Our present study included these same staff and added the new components described in this study as co-created booster training. Our goal was for 100% of the existing AHC team members to participate in this study and to use the SPAN framework to co-create the quality improvement plan.

## Methods

### Peer planning method

**Figure 1:**
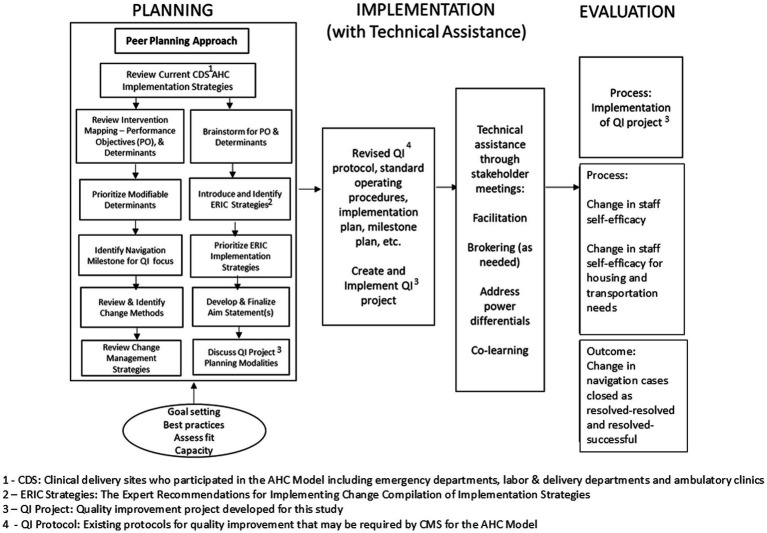
SPAN peer planning model.

The study was reviewed by the UTHealth IRB, protocol number HSC-SPH-20-0152. It was approved exempt. The peer planning approach for this study is shown in Figure 1. Briefly, the SPAN assessment and results have been reported elsewhere (7, 19). Our focus in this study was on pilot testing the SPAN model for peer planning, quality improvement and evaluation of impact on navigation milestones at one Bridge Organization in the AHC Model as shown in the Figure. SPAN is a triple loop learning model where organizations participate as co-creators through structured processes, and the technical assistance team as facilitators seek to shift not only organizational processes for navigation, but also their approaches to learning (26).

### Stakeholder committee meetings

As described in (19), a stakeholder committee was comprised of representatives including hospital, Bridge Organization, and research staff. The stakeholder committee was officially formed from an email invitation to all staff or leadership listed in the AHC Model implementation plan (required documentation) with an overview document describing the study and how to accept participation (via email confirmation). The history of the AHC project, objectives of the study, and purpose for future stakeholder and intervention mapping (IM) sessions was also shared with the team. The research team held a discussion to establish terms of the committee including meeting norms, vision, roles and responsibilities, decision-making processes and communication plans. As determined collectively, all decisions made by the stakeholder committee would be made via consensus with the use of voting and discussion following Robert’s Rules of Order. Finally, the committee was asked to input into the next meeting’s agenda. As part of every stakeholder meeting the team was asked if anyone else should join the committee to ensure all stakeholders were represented. Meetings were recorded and notes were taken by an assigned staff member during each meeting to create meeting minutes.

The second stakeholder meeting was held 2 weeks after the first meeting. Minutes from the previous meeting were reviewed and approved by the committee. A summary of results from the mixed methods assessment of current implementation strategies was presented to the committee. First baseline data on current implementation was reviewed, including: rates of HRSN screening, positives for HRSN, navigation acceptance rates and HRSN resolution from navigation services. Next, preliminary interview themes around barriers and facilitators to AHC Model implementation were checked to validate findings. Lastly, the process maps of current workflows for screening, referral and navigation were reviewed and edited to ensure accuracy. Since the IM peer planning sessions were scheduled to occur in the middle of the four stakeholder meetings, the structure and expectations were given for the planning sessions during this stakeholder meeting.

### Intervention mapping peer planning sessions

Prior to IM planning sessions, the research team gathered data from the SPAN assessment step (19) on the existing implementation process components and met and discussed the components that may be ready for enhancement or improvement, mapped the components to the theories underlying what would determine a change, and linked it to the ERIC strategies for consistency in terminology, understanding, and replicability.

Three, three-hour, IM Adapt peer planning meetings were held via WebEx. In the first meeting, the final interview themes and updated process map from the mixed assessment were first reviewed with the committee. The interview themes were linked to specific CFIR constructs and informed conversations about quality improvement opportunities. In addition, the research team reviewed current implementation strategies with the AHC clinical delivery site. In the second and third meeting, three separate breakout rooms for implementers (navigation staff/CHWs), managers, and leadership/IT staff were created. IM Adapt was used during the meeting to review current implementation strategies and barriers and facilitators in the AHC implementation (Meeting #2), brainstorm performance objectives and determinants (Meeting #3), and identify potential implementation strategies for performance objectives and determinants using the ERIC compilation (Meeting #3) (27). Each breakout room had an assigned facilitator who used open-ended questions to brainstorm performance objectives, determinants and implementation strategies. The key questions brainstormed were: Who are the implementers? What do they need to do? These first two questions represent performance objectives. Why would they do it? This question represents determinants. How should they do it? Is it feasible? These questions represent implementation strategies and change management. Responses from each of the breakout rooms’ participants were entered real-time into jamboard and an Excel spreadsheet (see Supplementary material) that was compiled by the research team into a single document for review and approval at a subsequent stakeholder meeting.

The result of these IM planning sessions process was a Quality Improvement Project template (see Supplementary material) with a theoretical foundation for success as shown by the equation below.

Performance Objectives + Determinants + ERIC Strategies = Quality Improvement Project Components.

An anonymous survey was emailed to the stakeholder committee members following the third and final peer planning session to give stakeholders the opportunity to prioritize the QI components identified during the planning sessions.

The third and fourth stakeholder meetings were held after the IM peer planning sessions. The QI components generated from the IM planning sessions and research team meeting were reviewed and summarized by the research team for the stakeholder committee. The research team facilitated discussion amongst the committee to ensure the performance objectives, determinants and ERIC strategies accurately represented the stakeholder’s perspectives. The committee then held a discussion to prioritize areas identified for potential quality improvement and change management strategies. During the fourth and final stakeholder meeting, an overview of designing a QI aim statement was provided and the team drafted the QI aim statement for their project. Finally, change management strategies were reviewed and linked to the implementation of the QI project. A smaller QI working group was formed from the stakeholder committee to develop the QI plan.

### Co-creation of quality improvement plan

To co-create a quality improvement plan, 5 h-long meetings were held via WebEx with the quality improvement working group. The first of QI team meeting began with a review of the QI Aim statement, determinants and change method to determine where the participants wanted to focus the QI plan. JamBoard was used during the discussion to provide a visual aid of how the change methods corresponded to the determinants being considered for the QI Project.

The second meeting began by reviewing the project QI aims and outcome. QI project planning began with the stakeholder committee and research team discussing possible training options for navigation staff. Consideration was given to the current training schedule for the navigation staff to ensure the additional training did not overburden the staff.

Having established the training constraints of the navigation staff, during the third QI project planning meeting, the research team was able to draft several training options with accompanying budgets for the stakeholder committee to review. The committee and research team discussed the pros and cons of each training option. Questions were generated from the stakeholder committee regarding the flexibility of the training options for the research team to take back to the training vendors.

During the fourth QI project planning meeting, the stakeholder committee and research team discussed the desired level of specificity and delivery method of navigation case management support complete with a demonstration. The QI Project evaluation was discussed, and QI charter protocol reviewed during the meeting. Lastly, the committee and research team finalized the QI project’s budget for submission to the selected vendors.

The final QI project planning meeting was used to review the finalized QI plan, QI project budget, and QI evaluation plan. Having gotten the approval from the stakeholders, research team, vendors and CMS, this was the final meeting before the launch of the QI project. This meeting was a time for managers to ask any lingering logistic questions and confirm the training’s scheduled dates.

### Training development

Once the committee settled on the QI plan of additional intensive training around the identified problem areas of housing and transportation navigation, the research team set out to compile a list of reputable vendors with subject matter expertise in the identified areas. The research team reviewed established collaborative connections and relationships with community organizations to identify potential partners. After fielding the potential partners for participation interest and time and resource availability to provide adequate training within the parameters of the project, two partners, one specializing in housing navigation and the other specializing in transportation navigation, with frontline experience were selected.

Once selected, the research team worked with vendors to develop and tailor content for training sessions to ensure synchronization with the prioritized determinants, strategies, and desired outcomes. Training sessions were then delivered in person and via WebEx.

### Evaluation

**Figure 2:**
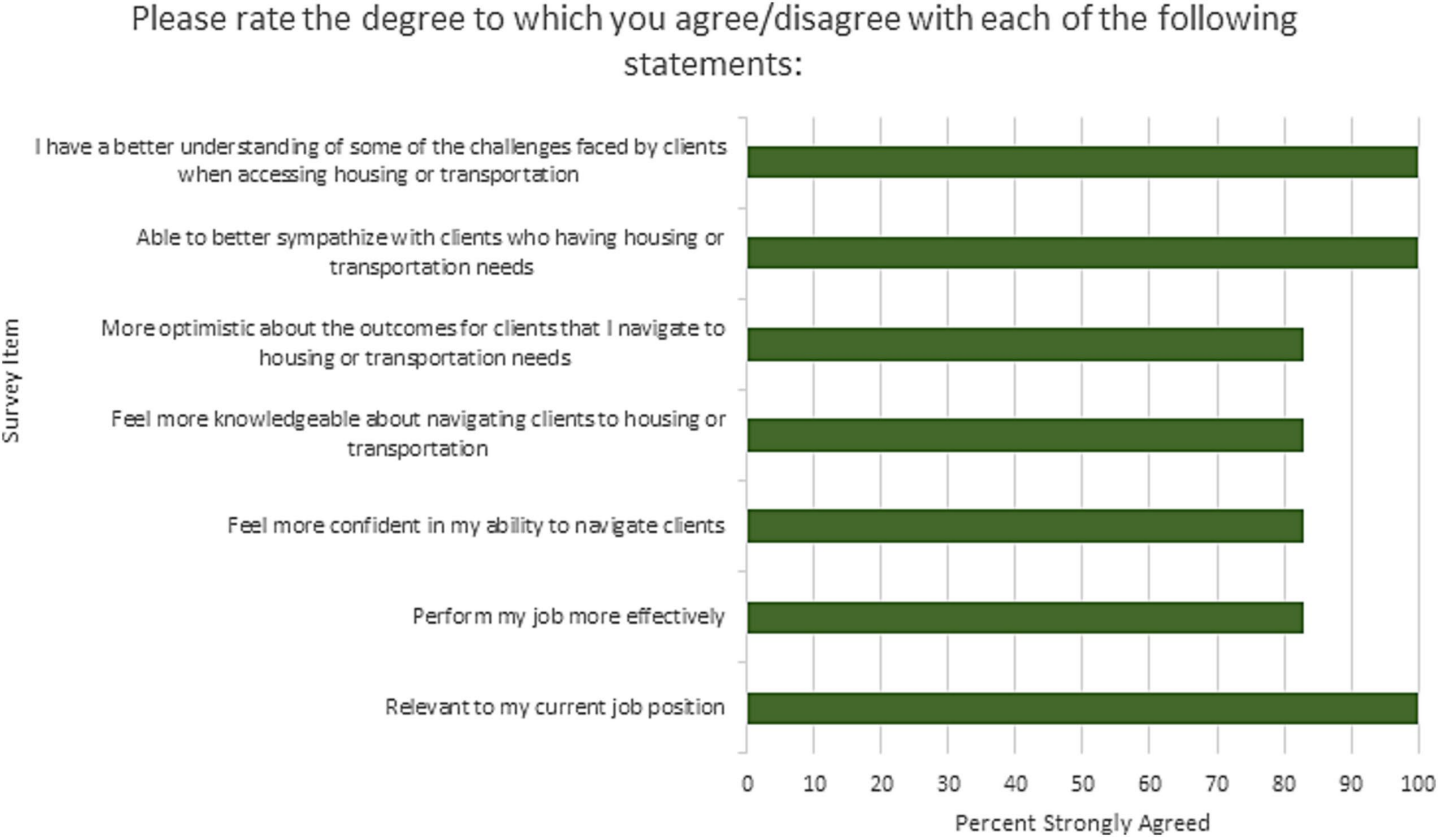
Percent agreement with survey items assessed.

Psychosocial outcomes were assessed at baseline and following training. Perceived self-efficacy was measured using a previously validated 12-item self-efficacy questionnaire (SE-12, Axboe et al. (32)) programmed in Qualtrics. The final item of the SE-12 was used as two separate statements with one word added for grammatical clarity to assess changes in navigation to housing and transportation resources, resulting in a 13-item survey (see Table 1, results). Navigators and managers were asked questions on the training curriculum and its impact on their knowledge, confidence, empathy for patient needs, optimism and relevance to their position in REDCap (see Figure 2, Results). Data on a selected client navigation outcome was used to assess the change in the number of navigation cases resolved from baseline (1 year) to 6 months post-training. The AHC Model navigation outcomes were Resolved: Resolved (beneficiaries’ need had been met), Resolved: Successful (the patient contacted the agency, and their need was believed possible to be addressed within 6 months), Unresolved: Unavailable (no community resource available to address the need for more than 6 months), Unresolved: Attempt Failed (the navigator made three unsuccessful outreach attempts to the beneficiary). The outcome measure for this study was a navigation case status of Resolved: Resolved or Resolved: Successful. A paired t-test was used to assess change from baseline.

**Table 1:** Self-efficacy survey results and baseline and post-training.

Self-efficacy Survey Item	Baseline		Post-Training		Difference
How certain are you that you are able to successfully ….	Mean (SD)	Median (IQR)	Mean (SD)	Median (IQR)	Median
1. Identify the issues the patient wishes to address during the conversation?	8.0 (2.09)	8.5 (4.0)	8.5 (2.07)	9.5 (3.0)	1.0
2. Make an agenda/plan for the conversation with the patient?	6.5 (2.66)	6.0 (4.0)	8.5 (2.35)	9.5 (2.0)	3.5
3. Urge the patient to expand on his or her problems/worries?	6.7 (1.97)	6.0 (3.0)	8.7 (1.97)	9.5 (2.0)	3.5
4. Listen attentively without interrupting or changing the focus?	8.8 (2.00)	10.0 (1.0)	9.2 (2.04)	10 (0)	0
5. Encourage the patient to express thoughts and feelings?	7.8 (1.94)	8.5 (3.0)	8.8 (1.47)	9.5 (3.0)	1.0
6. Structure the conversation with the patient?	7.7 (2.07)	7.5 (4.0)	8.0 (2.76)	9 (3.0)	2.5
7. Demonstrate appropriate non-verbal behavior (eye contact, facial expressions, placement, posture and voicing)?	8.2 (1.94)	8.5 (3.0)	9.2 (1.79)	10 (2.0)	1.5
8. Show empathy (acknowledge the patient’s views and feelings)?	8.4 (1.97)	9.0 (2.0)	8.5 (2.51)	9.5 (3.0)	0.5
9. Clarify what the patient knows in order to communicate the right amount of information?	8.2 (2.07)	9.0 (3.0)	8.7 (2.34)	10 (1.0)	1.0
10. Check patient’s understanding of the information given?	8.3 (1.97)	9.0 (3.0)	9.0 (2.00)	10 (1.0)	1.0
11. Make a plan based on shared decisions between you and the patient?	8.2 (1.94)	8.5 (3.0)	9.0 (2.00)	9.5 (1.0)	1.0
12. Navigate the client to helpful [transportation^*^] resources?	6.2 (3.55)	6.0 (5.0)	8.7 (1.97)	10 (2.0)	4.0
13. Navigate the client to helpful [housing^*^] resources?	6.0 (3.61)	5.0 (5.0)	8.7 (1.97)	9.5 (2.0)	4.5

* Added word for grammatical clarity. Growth in self-efficacy in navigating patients with housing and/or transportation HRSN specifically was the area with the largest improvement post-training (4-point increase), followed by urging the patient to expand on their concerns, structuring the conversation, and making a plan (see green boxes).

## Results

Representatives agreeing to participate in the stakeholder committee included executive leadership from the hospital ER navigation program (n = 2), Directors and Managers from the ER navigation program (n = 3), front-line patient navigators from the ER navigation program (n = 1), leadership, and staff from the AHC Bridge Organization (principal investigator, project manager; n = 2). Faculty and students with expertise in mixed methods and quality improvement led the meetings as external facilitators. A total of eight people comprised the stakeholder committee with 100% of the AHC team participating. In addition, the training developed in the study was open to all navigators and managers working at the partnering health system. An additional nine staff participated in training.

### Intervention mapping peer planning sessions

The performance objectives, determinants and ERIC implementation strategies brainstormed and prioritized by the team for the QI plan are shown in Table 2. The team decided to focus on the patient navigator and navigator manager roles for QI. Knowledge, self-efficacy, and outcome expectations were the selected behavioral determinants based on barriers identified in the assessment and stakeholder/planning meetings. ERIC implementation strategies were selected from the repository to match the prioritized determinants.

**Table 2:** Prioritized performance objectives, determinants and ERIC implementation strategies from the IM Planning Sessions.

Implementor	QI Performance Objectives	Prioritized Determinants	Current Barriers	ERIC Strategies Selected
Patient Navigator	Attend program training Complete navigation process post-training: Review priority list and work on patients based on that timeline. Review community resources with patient, what resources were sent, gather what resources patient may need and provide new resources as needed, use RedCap scripting, and document all encounters Become an expert with knowledge of local housing and transportation resources	Community resource knowledge Self-efficacy and skills to engage beneficiaries, assess and address concerns and needs Outcome expectations	Fear of being able to engage beneficiaries to talk about their needs. Knowledge of community resources Level of motivational interviewing skills	19. Intervene with Patients to Enhance Navigation process 23. Provide Ongoing Consultation 31. Capture and Share Local knowledge 32. Engage Community Resources
Navigation Manager	Support a culture of training by co-creating QI training plan and requiring staff to participate in continuing education Support staff by participating in training to become a team of experts in housing and transportation community resources Meet weekly with team to address goals, problems, and solutions Proactively identify navigation roadbloacks and solutions (e.g., patient rush off phone, set a plan to talk/identify barriers to talking/better time to call)	Budget/resources Self-efficacy Outcome expectations	Self-efficacy Time/effort	7. Provide Local Technical Assistance 20. Develop Educational Materials 21. Distribute Educational Materials 24. Conduct Ongoing Training

### Co-creation of quality improvement plan

The co-created QI plan focused on resolved navigation cases as the main outcome for QI. Three specific aims for training were developed to increase resolution of HRSN. 1) Conduct ongoing training focused on discussion, facilitation, active learning, consciousness raising, and technical assistance/capacity building to improve the knowledge of AHC navigators about housing and transportation resources in the City of Houston/Harris County to increase our percentage of resolved-successful navigation cases. 2) Conduct ongoing training focused on tailoring, modeling, participation, monitoring and feedback, and skill building/guided practice to improve the self-efficacy of AHC navigators to navigate patients to housing and transportation resources in the City of Houston/Harris County to increase our percentage of resolved-successful navigation cases. 3) Conduct ongoing training focused on elaboration and cues to action to improve the outcome expectations of AHC navigators to navigate patients to housing and transportation resources in the City of Houston/Harris County to increase our percentage of resolved-successful navigation cases.

### Implementation of didactic and hands-on training for navigation staff

The training curriculum included active learning, consciousness raising, elaboration, cues to action, tailoring, modeling, participation, monitoring and feedback, and skill building/guided practice to improve the knowledge, self-efficacy and outcome expectations of AHC navigators and managers to navigate patients to housing and transportation resources and to meet the specific aims from the QI plan. The training began with a half-day workshop for navigators focused on didactic training for skill-building. A total of 23 navigators attended. The workshop covered the following topics: diverse transportation navigation strategies and review of custom-tailored resources for transportation along with interactive exercises including case studies. Housing training included a demonstration of available resources, tips/tricks to navigation, and applied practice through case studies. A panel of community members with lived experience who have successfully navigated the system also presented their keys to success and challenges they encountered. Upon completion of the initial training, navigators were provided with necessary resources, including updated community resource directories. After training, they also met with the contractors biweekly for 6 months to collaboratively discuss, and problem solve challenging navigation cases with expert support, allowing for adaptive training and skill-building over time. A guidebook with challenging cases and proposed solutions was also developed to enhance skills practice and sustainability going forward. In addition, the contractors provided a monthly update on the capacity of each available community organization to resolve needs.

### Evaluation

23 navigators or managers completed the self-efficacy survey at baseline, 17 staff completed training and six completed the post-training survey (35% response overall). The six completing the post-training survey were all AHC staff and stakeholder committee members (75% response of those actively engaged in SPAN). Navigator self-efficacy results for those who completed both pre-post-surveys are shown in Table 1. The median change in SE score from baseline to post-training was 15 points, trending to an increase in self-efficacy. For individual items, all staff reported increases to conduct navigation activities assessed except for listening attentively which had a median score at baseline of 10 (highest). Growth in self-efficacy in navigating patients with housing and/or transportation HRSN specifically was the area with the largest improvement post-training (4-point increase), followed by urging the patient to expand on their concerns, structuring the conversation, and making a plan (see green boxes Table 1). As shown in Figure 2, 100% of survey respondents strongly agreed that the training had increased their knowledge and empathy for patients with housing and transportation needs. 100% strongly agreed the training was relevant to their job. 80% strongly agreed that the training increased their confidence, knowledge, and optimism to navigate patients overall and for these needs. The number of navigation cases with HRSNs coded as resolved during the baseline year (August 2020–August 2021) was an average of 758 cases per month. The number of navigation cases coded as resolved during the post-training period (Sept 2021-February 2022) was an average of 975 cases per month, an increase of 29% of cases resolved per month (*p* = 0.001).

## Discussion

The SPAN peer planning process piloted in this study led to the creation and implementation of a quality improvement plan and booster training for patient navigators and managers for HRSN navigation, with a particular focus on housing and transportation. As shown in the results, we found directional increases in navigator self-efficacy post-training, though due to limited sample size we were not able to test this change statistically. Due to the PHE, we used an online survey, yielding a response rate of 35% overall and 75% for staff actively engaged in SPAN. While lower than pre-PHE survey response rates, recent studies have shown similar PHE response rates (28, 29). We recommend readers interpret the survey results accordingly. The number of resolved HRSN cases over the following 6 months increased significantly from the baseline period. To our knowledge, this is the first study to report on methods for creating and implementing adaptive navigator training for certified CHWs working as professional patient navigators and managers specifically for HRSN in the hospital setting. Previous studies of HRSN navigation included peer to peer and student models (30, 31). These are similar to the Texas CHW generalist certification in competencies covered (30, 31). Our assessment indicated that additional training was needed for housing and transportation navigation. We found that providing adaptive and just in time training was helpful (26). Using peer planning and co-creation to identify specific skills foci for training with the front-live staff allowed for the creation of detailed learning objectives. In addition, the use of the bi-weekly meetings with housing and transportation experts provided the opportunity for adaptive and ongoing learning (26). While not specifically measured in this study, we found anecdotally that the time needed to support navigators decreased over time in these meetings which we hypothesize was due to increased self-efficacy. In the future, it would be helpful to measure this specifically. In addition, providing navigation services during the evolving PHE was challenging, even when using a structured approach to increase workforce and organizational capacity. Some factors that impact HRSN resolution are beyond the control of health systems, staff, facilitators and external experts. We found ongoing changes in community resource availability due to the PHE and had to regularly update the community resource directory for accuracy. At the policy level, Houston’s implementation of the end of the eviction moratorium created challenges to housing resolution with large increases in need coupled with dwindling housing availability. Another challenge to HRSN navigator workforce development in the US is that all States all have different credentialling requirements, and there is no national body focused on the development of the HRSN workforce, particularly for link workers. These challenges highlight the impact of policy and the need for a systems approach that is integrated from the front-line to policy to support HRSN resolution.

## Data Availability

The raw data supporting the conclusions of this article will be made available by the authors, without undue reservation.
