# Supplementary material for: Co-creation of staff training to address health-related social needs in emergencies
Source: Front Public Health. 2025 Apr 9;13:1441368. doi: 10.3389/fpubh.2025.1441368 (PMC12014777; doi:10.3389/fpubh.2025.1441368)
Supplement: Supplementary file 1 [file Table_1.docx]

| **Bridge Organization Name:** |
| --- |
| ***QI Project Aim (Template):*** *To improve AHC navigation,...*  QI Project Aims:   1. To improve AHC navigation, the [insert person] will… 2. To improve AHC navigation, the [insert person] will.. 3. To improve AHC navigation, the [insert person] will... |
| **Bridge Organization QI Project Sponsor:** |
| **Project Background:**  The Accountable Health Communities (AHC) model was designed to address the health-related social needs of Centers for Medicare & Medicaid Services (CMS) beneficiaries. Bridge organizations across the AHC model have identified lack of technical assistance and peer planning as potential barriers to model success, particularly around patient navigation. The technical assistance and peer planning literature lacks an organizing, conceptual framework, but implementation science frameworks could serve as useful guides. The Strengthening Peer AHC Navigation (SPAN) project seeks to fill this gap and will apply three implementation science frameworks, Consolidated Framework for Implementation Research (CFIR), Intervention Mapping (IM), and the Expert Recommendations for Implementing Change (ERIC) compilation, to develop a multi-level quality improvement intervention. The aims of the SPAN project are to implement and evaluate a new multi-level QI intervention to improve AHC implementation and navigation milestones through structured peer planning and to provide successful technical assistance for the AHC model. |
| QI Project Scope: Navigation Action Plan   1. Choose one of the AHC Navigation Measures as the focus of the QI Plan |
| **QI Project Selected Implementation Strategies (take from CDS implementation table (see supplementary materials)):**   1. *Facilitation: Provide [activity] to [staff person]*   **Name the Implementers:**  a). Staff Person 1  b). Staff Person 2  **Specify the Determinants:**  Staff Person 1  Determinants  Staff Person 2  Determinants:  **Specify the ERIC Strategies** *Staff Person 1*  **5** Funds & Contract (bring in contractor or facilitator to help)  **20** Develop educational materials  **24**  Conduct ongoing training  *Staff Person 2*  **22** Conduct Educational Outreach, Visits  **23**  Provide Ongoing Consultation |
| **QI Project Change Management Strategies (take from planning meetings):**   1. Assess readiness for change    - Does this QI project align with our AHC and institutional goals?    - What do we need to prepare for the QI project implementation?    - What additional support(s) might you need as the QI team to successfully achieve the aims?    - What engagement do we need from our institutional leaders? 2. Establish a sense of urgency for change    - Why are we wanting to implement change?    - How can your team create a “burning platform,” in other words, an extremely urgent or compelling case to convey, in the strongest terms, the need for change?    - How can your team “make the case” to garner organizational support the change? 3. Assemble the steering team    - What are the QI project aims?    - Who is on the QI project team?    - What are the roles and responsibilities of everyone on the QI project team?    - Who is our program champion? 4. Create an implementation plan    - Budgets    - Step-by-step plans    - Implementation schedule    - Communication plans    - Elevator speech    - Feedback mechanisms    - Contingency plans    - Observation plans    - Strategies    - Short-term and long-term goals (Outcome and Process measures)    - Possible process measures 5. Pilot testing of implementation plan    - Do we want to pilot test? How would we pilot test?    - Plan Do Study Act 6. Disseminate change    - What created successful outcomes and what did not?    - What steps or elements must be changed prior to implementation? 7. Sustainability plan    - How do we sustain the change?    - How do we sustain the successful outcomes? |
| **Estimated Date for Start and End of QI Project:**  Preparation and planning:  QI Implementation and Measurement: |
| Team Meeting Frequency during QI Project: |
| Team Members Participating: |
